# National Emergency Resuscitation Airway Audit (NERAA): a pilot multicentre analysis of emergency intubations in Irish emergency departments

**DOI:** 10.1186/s12873-022-00644-2

**Published:** 2022-05-28

**Authors:** Etimbuk Umana, James Foley, Irene Grossi, Conor Deasy, Francis O’Keeffe, and on behalf of Irish Trainee Emergency Research Network (ITERN) Collaborators, Andrew Patton, Marcus Jee, Jeffery Mulcaire, Ahmed Al Rasheed Ahmed, Claudio Dalla Vecchia, Nikita Vainberg, Hugh O’ Reilly, Joseph Daly, Cathal de Buitléir, Conor Prendergast, Randa Ibrahim, Shane Kukaswadia

**Affiliations:** 1 Department of Emergency Medicine, Connolly Hospital Blanchardstown, Mill Road, Abbotstown, Dublin, Ireland; 2 Irish Trainee Emergency Research Network (ITERN), Dublin, Ireland; 3 Department of Emergency Medicine, University Hospital Waterford, Waterford, Ireland; 4 Department of Emergency Medicine, University Hospital Limerick, Limerick, Ireland; 5 Department of Emergency Medicine, Cork University Hospital, Cork, Ireland; 6 Department of Emergency Medicine, Mater Misericordiae University Hospital, Dublin, Ireland

**Keywords:** Airway management, Emergency department, Rapid sequence induction, Intubation

## Abstract

**Background:**

There is paucity of literature on why and how patients are intubated, and by whom, in Irish Emergency Departments (EDs). The aim of this pilot study was to characterise emergency airway management (EAM) of critically unwell patients presenting to Irish EDs.

**Methods:**

A multisite prospective pilot study was undertaken from February 10 to May 10, 2020. This project was facilitated through the Irish Trainee Emergency Research Network (ITERN). All patients over 16 years of age requiring EAM were included. Eleven EDs participated in the project. Data recorded included patients’ demographics, indication for intubation, technique of airway management, medications used to facilitate intubation, level of training and specialty of the intubating clinician, number of attempts, success/complications rates and variation across centres.

**Results:**

Over a 3-month period, 118 patients underwent 131 intubation attempts across 11 EDs. The median age was 57 years (IQR: 40–70). Medical indications were reported in 83% of patients compared to 17% for trauma. Of the 118 patients intubated, Emergency Medicine (EM) doctors performed 54% of initial intubations, while anaesthesiology/intensive care medicine (ICM) doctors performed 46%. The majority (90%) of intubating clinicians were at registrar level. Emergency intubation check lists, video laryngoscopy and bougie were used in 55, 53 and 64% of first attempts, respectively. The first pass success rate was 89%. Intubation complications occurred in 19% of patients. EM doctors undertook a greater proportion of intubations in EDs with > 50,000 attendance (65%) compared to EDs with < 50,000 attendances (16%) (*p* < 0.000).

**Conclusion:**

This is the first study to describe EAM in Irish EDs, and demonstrates comparable first pass success and complication rates to international studies. This study highlights the need for continuous EAM surveillance and could provide a vector for developing national standards for EAM and EAM training in Irish EDs.

**Supplementary Information:**

The online version contains supplementary material available at 10.1186/s12873-022-00644-2.

## Background

Emergency airway management (EAM) is an integral component in the management of critically unwell patients presenting to the emergency department (ED). Internationally, there is significant variation in personnel and practices around rapid sequence induction (RSI) and intubation of patients in the ED [1–6]. This variation in terms of personnel and practice also extends to large and small EDs undertaking EAM [1]. This high-risk procedure is performed by both anaesthesiology/intensive care medicine (ICM) and emergency medicine (EM) doctors in the ED and should be undertaken safely. ED airway registries exist in jurisdictions including Australia, Japan, and the USA [1, 3, 7]. The National Emergency Airway Register (NEAR) in the USA has been collating data for over a decade [7]. These registries have been developed to undertake active surveillance of EAM for quality control and improvement. They support the understanding of current practices, trends, guideline development and implementation.

EAM is not without complications which can range from hypoxemia to cardiac arrest [1, 3–5, 7, 8]. In the UK, the 4th National Audit Project of the Royal College of Anaesthetists and The Difficult Airway Society (NAP4) was published in March 2011 [9]. The NAP4 found that, among 15 severe complications associated with airway management in the ED, the majority occurred out of hours, without senior supervision, or without the operators following standard airway management algorithms [9]. This led to important recommendations throughout the UK for EAM in EDs [8, 9]. Some of these recommendations included: performing airway risk assessments, use of capnography and intubation checklists, joint training for EM and anaesthesiology/ICM staff, and regular audits of EAM [8].

Although EAM is frequently performed in Irish EDs today, there is a paucity of literature on why and how patients are intubated, and by whom. Traditionally, EAM was undertaken in Ireland by anaesthesiology/ICM doctors in EDs. However, with the advent of EM training in Ireland over the last two decades, EM doctors now play a very active role in EAM in EDs. Understanding the current practice of EAM and variation between sites in Ireland is essential for improving patient safety, staff education and clinical practice development. The aim of this study was to characterise EAM of critically unwell patients in Irish EDs with specific focus on indications, staffing, airway techniques, complications, success rates and variations across different centres.

## Methods

National Emergency Resuscitation Airway Audit (NERAA) is a prospective multicentre study of RSI and intubation practice for critically unwell patients in EDs throughout Ireland. NERAA was conducted as a pilot study to test the feasibility of establishing a national airway registry for EDs in Ireland (Emergency Medicine Airway Registry Ireland (EMARI)). This study has been reported in conformity with the Strengthening the Reporting of Observational Studies in Epidemiology (STROBE) standards [10].

### Study setting and participants

NERAA is the first project performed by the newly established Irish Trainee Emergency Research Network (ITERN) following the success of the Trainee Emergency Research Network (TERN) in the UK [11]. The ITERN framework is made up of a central committee with co-investigators (CI) across the country. The network members are non-consultant hospital doctors (NCHDs) of mostly registrar level but also includes senior house officers (SHOs).

There are 29 EDs in Ireland, 3 are paediatric only, 5 are adult only while the remainder are mixed. Sixteen EDs are accredited training centres for EM. Of the 26 mixed and adult EDs, 11 sites registered to participate in NERAA and obtained approval prior to the study onset. These 11 EDs were all considered type A, defined as 24-hour consultant led EDs with resuscitation and critical care availability. Of the 11 EDs, 9 were training sites for EM. The ED attendances ranged from 25,000 to 82,000 patients per year. Intubation is undertaken by either EM or anaesthesiology/ICM doctors from SHO to consultant level in these EDs. Junior doctors or NCHDs are a mixture of both training and non-training positions. SHOs are post internship doctors working from PGY 2–4, registrars (not in training) are PGY 3–5, specialist registrar (on training) are PGY 4–8 and fellows are PGY 8 and above undertaking post completion of training subspecialisation that can last up to 2 years before becoming consultants.

Patients over the age of 16 years with an attempted intubation in participating EDs were identified and included during a 3-month period from February 10 to May 10, 2020.

### Data collection and processing

The intubating clinician at each site entered the procedural data onto the EMARI proforma (Supplement 1). This proforma was implemented for use in all 11 EDs participating in the study. This document was then placed in a secure box. Patients were assigned a case number for each hospital. The patients’ medical record number was known to the CI and was only used if they needed to retrospectively complete missing data or complete unfilled proformas. CIs cross referenced data with ED and Intensive Care Unit (ICU) records to mitigate missed patients. The EMARI proformas were collected by the CI at each site every week and anonymised data uploaded onto a secure Research Electronic Data Capture (REDCap™) online platform with no patient identifiers. REDCap™ is compliant with the Good Clinical Practice and the European General Data Protection Regulation on data management [12, 13]. Access to REDCap™ was provided by University College Dublin (UCD) Clinical Research Centre (CRC). UCD CRC institution did not formally take part in the study; they provided access to REDCap™ only. From March 17, 2020 onwards there was a change in intubating procedures due to COVID19, with EAM described as an aerosol generating procedure. On March 24, 2020 Ireland went into a national lock down which saw significantly reduced numbers presenting to EDs [14].

Data was collected on patient age, gender, indication for intubation, technique of airway management, names and dosages of all medications used to facilitate intubation, grade of training and specialty of the intubating clinician, number of attempts, success or failure, and complications. Data definitions were based on EAM studies by Fogg et al. and Alkhouri et al. [1, 15]. An attempt at intubation was defined as single passage of a laryngoscope into the mouth with successful passage of the endotracheal tube through the vocal cords defined as a successful intubation. A difficult intubation was defined as requiring 2 or more attempts. Complications were pre-defined and included equipment failure, hypotension requiring fluids or inotropes, desaturation (SpO2 less than 93%), bradycardia (heart rate less than 60), dental or airway trauma due to intubation attempt, second dose of paralytic required, oesophageal or mainstem bronchial intubation, vomiting, laryngospasm, medication error or cardiac arrest.

### Statistical analysis

Results are reported as proportions for categorical variables and medians with inter quartile range (IQRs) for continuous variables. Chi squared and fisher’s exact tests were used to determine associations between categorical variables with missing data excluded in the analysis. To evaluate variation, EDs were grouped into those greater than 50,000 (> 50 K) versus less than 50,000 attendances per year (< 50 K). A consensus decision was agreed by the investigators that 50 K attendances cut off was a good differentiator between EDs in Ireland with high volume attendances vs low volume. Sites with higher attendances generally have a larger volume of airway trained EM and anaesthesiology/ICM doctors. In addition, this cut off would be a good discriminator to assess for variation in EAM practice. For data analysis, we used Statistical Package for Social Sciences (SPSS version 23, IBM, USA). A *p* value of less than 0.05 was considered statistically significant.

### Patient and public involvement

Patients or the public were not involved in the design, or conduct, or manuscript production of our pilot study.

## Results

### Patient demographics, indications for intubation and pre-intubation vital signs

During the study period there were 109,738 presentations to the 11 participating EDs, 131 intubation attempts were recorded for 118 patients. In the first month of the study 44 (37%) patients undergoing EAM had been recorded, while in month 2 and 3 it was 44 (37%) and 30 (26%) respectively (supplement 2 reports EAM recorded per week and ED attendance per month). Post the implementation of EAM as aerosol generating procedure due to COVID19 (March 17th), 55 (47%) patients underwent EAM in the ED. Thirty (25%) patients were aged between 18 and 40 years, 37 (31%) between 41 and 60 years and 51 (44%) were greater than 60 years. Females accounted for 41% of intubated patients. Medical indications were reported in 98 (83%) patients compared to 20 (17%) for trauma. Indications for intubation are summarised in Table 1. The most common medical indication was cardiac arrest [29 (30%)] with 79% undertaken by EM doctors. The majority (85%) of trauma patients were seen in EDs with > 50 K attendances.

**Table 1:** Demographics, indications, vitals, staffing and drugs

Variable	n (%)
Age, median (Interquartile range)	57 (40–70)
Gender (Male)	70 (59)
Weight, median (Interquartile range) ^a^	75 (70–87)
Indication: Medical	98 (83%)
Respiratory failure ^c^	17 (18)
Anaphylaxis	1 (1)
Cardiac failure	2 (2)
Sepsis	2 (2)
Seizures	10 (10)
Altered Mental Status	9 (9)
Overdose/poisoning	14 (14)
Cardiac arrest	29 (30)
Intracranial haemorrhage/stroke	14 (14)
Indication: Trauma	20 (17%)
Traumatic cardiac arrest	2 (10)
Neck/facial trauma	2 (10)
Chest trauma	2 (10)
Head injury- airway not patent	1 (5)
Head injury- threatened airway	13 (65)
Vitals Pre-Intubation at time of decision to intubate (Non cardiac arrest) ^b^	
Glasgow coma scale (< 9)	59 (66)
Systolic blood pressure (< 90)	11 (12)
Heart rate (> 100)	31 (35)
Oxygen saturation -SaO2 (< 93%)	26 (29)
Intubating Clinician Grade	
Consultant	7 (6)
Fellow	1 (1)
Specialist Registrar (SpR)	45 (38)
Registrar	61 (52)
Senior House Officer (SHO)	4 (3)
Drugs Induction	
Propofol	59 (50)
Ketamine	19 (16)
Midazolam	25 (21)
No sedation	27 (23)
Drugs Muscle relaxant	
Rocuronium	87 (74)
Suxamethonium	7 (6)
No muscle relaxant	24 (20)

^a^N-88 patients had weight recorded

^b^N-89 non cardiac arrest patients. No patient was intubated for airway obstruction or GI bleed for medical indications. For trauma indications no patient was intubated for shock, burns/inhalation, drowning or penetrating trauma

^c^Nine (8%) patients were intubated for COVID19 due to respiratory failure

### Staffing, drugs, and intubation success

**Fig. 1:**
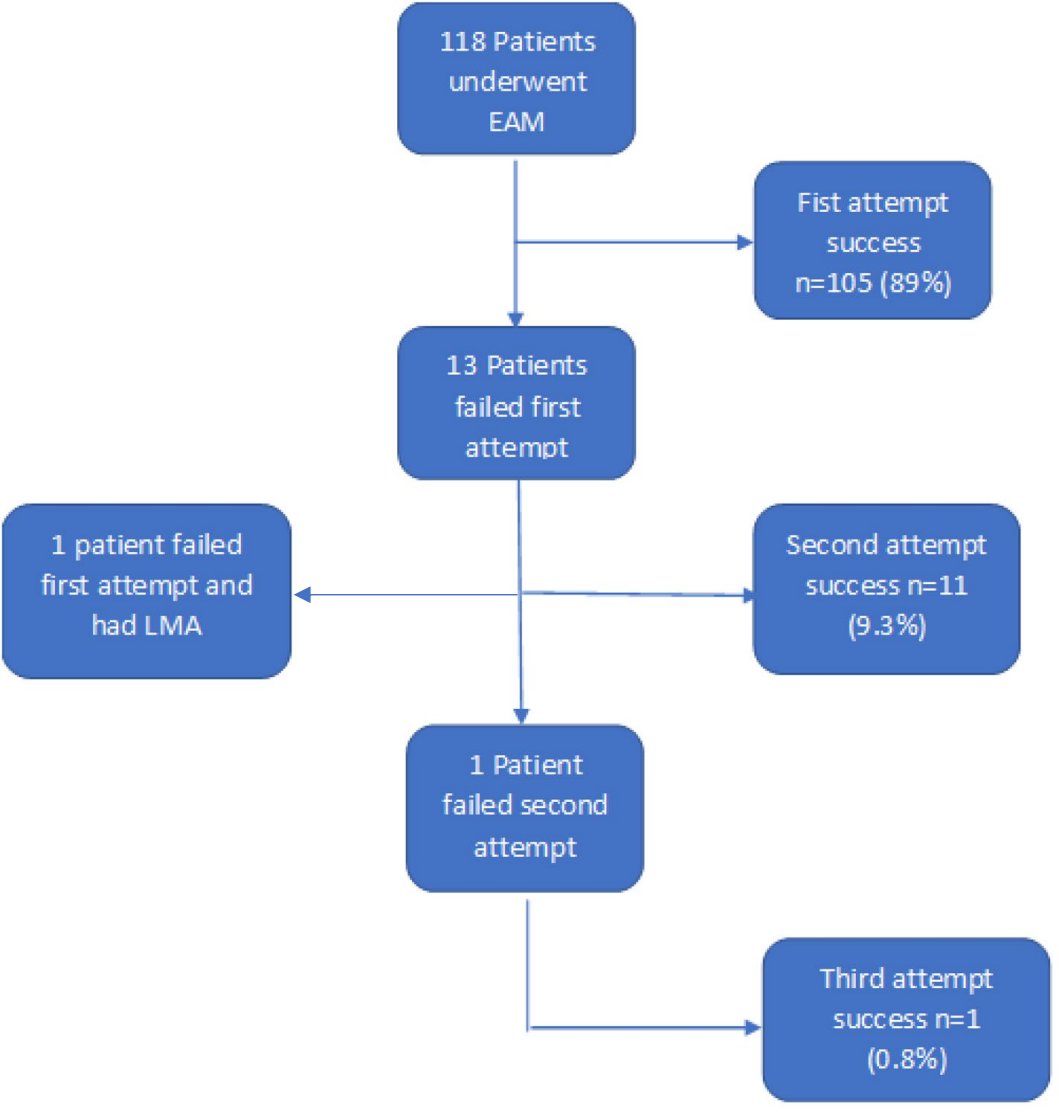
Flow chart of intubation attempts. (EAM – emergency airway management, LMA – laryngeal mask airway)

EM doctors performed 64 (54%) of the initial intubations, while anaesthesiology/ICM doctors performed 54 (46%) from 118 patients. A breakdown of grades of doctors undertaking initial intubation is displayed in Table 1. The most common induction drug was propofol [59 (50%)], while rocuronium was the most common [87 (74%)] paralytic used for intubations (Table 1). There were 15 (13%) patients who had a combination of midazolam with either propofol or ketamine. Of the 29 medical cardiac arrest patients intubated, 13 (45%) patients had paralytics given. No patient in traumatic cardiac arrest had paralytics. Total successful intubations occurred in 117 (99%) patients on 3 attempts. Figure 1 displays the flow chart of intubation attempts. A patient in cardiac arrest at presentation had a single failed attempt at intubation and had a laryngeal mask airway (LMA) subsequently inserted for ongoing EAM.

### Pre-intubation: airway assessment, pre-oxygenation and patient position

A documented airway assessment was made in 87 (74%) patients with 30 predicted to have a difficult airway. An intubation checklist was used in 65 (55%) patients. EM doctors [42 (66%)] were more likely to use an intubation checklist compared to anaesthesiology/ICM doctors [23 (43%)] (*P* = 0.012). Also, an intubation checklist was used in 48% of cardiac arrest patients. The most common pre oxygenation device used was a bag valve mask (BVM) with a positive end expiratory pressure (PEEP) valve in 67 (57%) patients. Apnoeic oxygenation was undertaken in 35 (30%) of initial intubation attempts with BVM [15 (13%)] being the most common strategy utilised. The majority [76 (65%)] of patients were positioned flat for intubation (Table 2).

**Table 2:** PRE-Intubation: airway assessment, pre-oxygenation, and patient position

Variable	ED > 50 K attendances-93 n (%)	ED < 50 K attendances-25 n (%)	*P*-Value
Airway assessment made			0.825
Yes	69 (74)	18 (72)	
No	24 (26)	7 (28)	
Difficult airway predicted			< 0.000
Yes	30 (32)	0 (0)	
No	39 (42)	17 (68)	
Not documented	24 (26)	8 (32)	
Emergency intubation checklist completed			0.371
Yes	58 (62)	18 (72)	
No	35 (38)	7 (28)	
Preoxygenation (Final device used)			0.012
NRBM	9 (10)	5 (20)	
BVM	15 (16)	6 (24)	
BVM + PEEP	55 (59)	12 (48)	
NIV	1 (1)	1 (4)	
LMA	13 (14)	0 (0)	
None	0 (0)	1 (4)	
Apnoeic Oxygenation			0.078
NP	11 (12)	3 (12)	
BVM	8 (9)	7 (28)	
NP + BVM	3 (3)	1 (4)	
NIV	1 (1)	1 (4)	
None	70 (75)	13 (52)	
Patient Position			0.045
Flat	55 (59)	21 (84)	
Pillow or occipital pad	14 (15)	3 (12)	
Bed tilted head up	17 (18)	1 (4)	
Ramped or head up	7 (8)	0 (0)	

*BVM* Bag Valve Mask, *LMA* Laryngeal Mask Airway, *NRBM* Non Re-Breather Mask, *NIV* Non-Invasive Ventilation, *NP* Nasal Prongs, *PEEP* Postive End Expiratory Pressure

### During intubation: intubating clinicians, intubation equipment and manoeuvres

Anaesthesiology/ICM doctors had a first pass success rate of 94%, while EM doctors first pass success was 86%. There was no statistically significant difference between anaesthesiology/ICM and EM doctors in terms of first pass success rate (*P* = 0.120). Of the intubating clinicians, 81 (69%) had performed > 100 previous intubations, while 34 (29%) had performed between 10 and 100 previous intubations with 3 (2%) having performed < 10 previous intubations. In terms of laryngoscopy, 62 (53%) patients had a video laryngoscope used on initial intubation attempt. A Cormack & Lehane grade I and II was reported in 85 (72%) initial intubation attempts (Table 3).

**Table 3:** INTRA-intubation: intubating clinician, intubation equipment and manoeuvres

Variable	ED > 50 K attendances-93 n (%)	ED < 50 K attendances-25 n (%)	*P*-Value
Intubating Clinician Specialty			< 0.000
Emergency Medicine	60 (65)	4 (16)	
Anaesthesiology/ICM	33 (35)	21 (84)	
Laryngoscopy			0.937
Macintosh	43 (46)	11 (44)	
Video	49 (53)	13 (52)	
Not documented	1 (1)	1 (4)	
Cormac & Lehane			0.253
Grade I	49 (53)	15 (60)	
Grade II	16 (17)	5 (20)	
Grade III	3 (3)	0 (0)	
Grade IV	5 (5)	0 (0)	
Not documented	20 (22)	5 (20)	
Introducer			0.002
Neither	25 (27)	9 (36)	
Bougie	65 (70)	10 (40)	
Stylet	3 (3)	5 (20)	
Not documented	0 (0)	1 (4)	
External Laryngeal Manipulation			0.822
Yes	14 (15)	3 (12)	
No	76 (82)	19 (76)	
Not documented	3 (3)	3 (12)	
Cricoid			0.303
Yes	14 (15)	6 (24)	
No	74 (80)	18 (72)	
Not documented	5 (5)	1 (4)	

*ICM* Intensive Care Medicine

### Post-intubation: tube placement confirmation, complications, outcome and variation

**Fig. 2:**
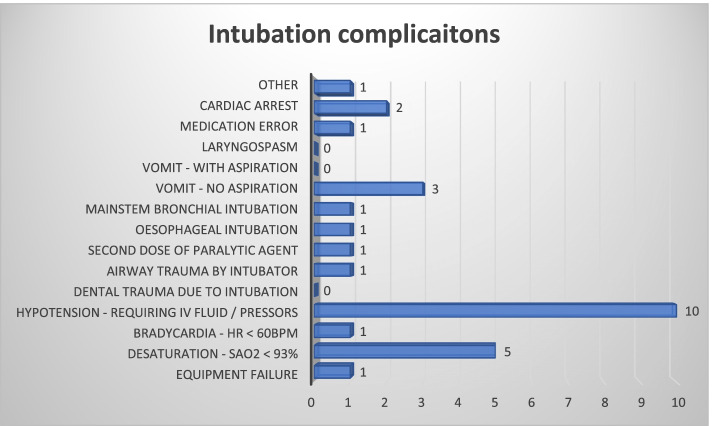
Intubation complication [23 patients (19%)]. Other = Cardiac arrest patient with failed first attempt and LMA (laryngeal mask airway) subsequently inserted for remainder of arrest (Patient died in ED)

Endotracheal tube placement confirmation was via waveform capnography in 114 (96%), while 3 (3%) had confirmation with clinical confirmation alone and 1 had no record of confirmation. Of the 4 cases with no capnography confirmation 2 cases were undertaken by EM doctors while the remainder were by anaesthesiology/ICM doctors. Intubation complications were reported in 23 (19%) patients, with 4 patients having 2 or more complications. There was no significant difference in complications rates between EM doctors [14 (22%) and anaesthesiology/ICM doctors [9 (17%)] (*P* = 0.477). The complications are displayed in Fig. 2. No patient required cricothyroidotomy for rescue after a failed intubation attempt. Table 4 shows the outcome of all patients following intubation. The variation between EDs with > 50 K compared to < 50 K attendances observed were, reported difficult airway predicted, preoxygenation device, patient position, intubating speciality and introducer used.

**Table 4:** POST-intubation: tube placement confirmation, complication, and outcome

Variable	ED > 50 K attendances-93 n (%)	ED < 50 K attendances-25 n (%)	*P*-Value
ETT Placement Confirmation			0.235
Waveform capnography	90 (97)	24 (96)	
Clinical confirmation alone	3 (3)	0 (0)	
Not documented	0 (0)	1 (4)	
Intubation Complications			0.620
Yes	19 (20)	4 (16)	
No	74 (80)	21 (84)	
Outcome			0.050
ICU	64 (69)	18 (72)	
Theatre/Cath lab	4 (4)	0 (0)	
Transferred to another hospital	7 (8)	4 (16)	
Extubated in ED	0 (0)	1 (4)	
Died in ED	18 (19)	2 (8)	

*ED* Emergency Department, *ETT* Endotracheal Tube, *ICU* Intensive Care Unit

## Discussion

In this first multicentre analysis of EAM across Irish EDs, we report a first pass success rate of 89 and 19% complication rate. The first pass success rate in our study compares favourably with other multicentre EM intubation studies which range from 70.8–94% [1–3, 7, 16, 17]. A systematic review and metanalysis on ED intubations by Park et al. revealed 84% to be the benchmark for first pass success rate [18]. Though our first pass success rate is 89%, which is higher than the benchmark set by Park et al. [18], there is scope for improvement. Achieving first pass success is paramount, due to increased complication rates associated with multiple attempts [3, 5, 19]. Sakles et al. implemented a continuous quality improvement (QI) program in a single centre in the U. S which included ongoing intubation surveillance, educational training and EAM standardization over a 10-year period [20]. They found that the QI initiative resulted in improvement of the first pass success rate from 73.1 to 92.4% [20]. A more recent Australian single centre study demonstrated similar findings, with an increase in first attempt success rates from 88.5 to 94.6% after introduction of a QI bundle which comprised monthly airway management audit, education and an online airway management checklist [21]. These buttress the need for an ongoing airway registry and continued educational programs for training EM and anaesthesiology/ICM doctors involved in EAM across Irish hospitals. Such initiatives would result in improved first pass success rates, and better patient outcomes.

International airway registries report complication rates ranging from 6 to 26% [1, 3, 4, 7]. Our study had a complication rate of 19% which is closer to the higher range of previous studies. Patients with 2 or more intubation attempts also had a higher complication rate compared to 1 intubation attempt at success. This is in line with previous studies [1, 19], with one study reporting an incidence of 53.1% of complications in cases requiring more than 2 attempts at intubation [19]. Hypotension and desaturation were the most common complication reported in our study. Our findings were in contrasts with the Australian registry with similar classification and reporting of complications [1]. It is worth noting that our cohort had higher proportion of hypoxic (29%) and hypotensive (12%) patients compared to Alkhouri et al. (hypoxia − 17% and hypotensive- 11%) [1]. In their study, the reported desaturation and oesophageal intubation as the two most common complications [1]. This difference highlights the importance of having an airway registry which can inform areas of practice improvement, particularly around physiological optimisation of patients haemodynamics prior to intubation as evidenced in our study. Decreasing the rate of complications should also be a major focus. With the implementation of QI initiatives as previously described, a drop was noted in the complications by both Sakles et al. and Foggs et al. of 7.9 and 9.6% respectively [20, 22].

EM doctors performed 54% of all initial intubations with no significant difference in first pass success and complication rates in our study compared to anaesthesiology/ICM doctors. They mostly comprised of registrars with the majority having experience of > 100 previous intubations. This contrasted with the UK census study which revealed approximately 20% of intubations were performed by EM doctors [23]. However, this study was conducted back in 2010 and trends could have very well changed since that time. On the other hand, in other regions such as Japan, Australia and the U. S, EM led intubations range from 82.6–95% [1, 3, 7]. The higher rates of EM clinician led intubations, could be attributed to development and evolution of the specialty of EM in these countries. EM has been established and recognised as a specialty in USA and Australia, since the early 1980s [24]. Other factors contributing to this difference could be staffing ratios and financial incentives to undertake EAM in those jurisdictions.

EM in Ireland however is a young specialty with the recognition of Accident and Emergency Medicine as a specialty by the Irish Medical Council in 1997, to the appointment of the first two specialist registrars in EM in 2001. At this point (2001) in the Irish health system, there were only two full time consultants in EM appointed nationally. Since then, the training program has evolved and improved and incorporated airway training on anaesthesiology rotations. As EM continues to grow in Ireland, EAM is clearly becoming a fundamental skill in managing critically unwell patients. We noted from our results that EM doctors were more likely to undertake intubations in EDs with > 50 K attendances while aneasthesia/ICM doctors undertook most intubations in EDs with < 50 K attendances. This may explain the difference in practice between EDs with < 50 K and > 50 K attendances as highlighted in the results. These factors were not further assessed in this study and would be beyond the scope this project. However, further research would be needed to delineate these differences.

EAM checklist use in our study was 55%, which was similar to findings by Alkhouri et al. (59.7%) [1]. Although current evidence in the use of checklists is not associated with survival in the setting of EAM, their use assists with cognitive offloading, thereby reducing stress and errors [22, 25–27]. Hence, incorporating checklists should be encouraged as a safety mechanism for EAM. Utilisation of bougie and video laryngoscope were at 64 and 53% respectively. Both have been shown to be associated with improved first pass success rate, while video laryngoscopy improves glottic visualization [1, 28, 29]. Therefore, both the Difficult Airway Society (DAS) and Irish Association for Emergency Medicine (IAEM) guidelines on intubation recommend video laryngoscopy be available in every ED [30, 31]. These guidelines also recommend the use of a bougie on initial intubation attempt to maximise the chances of first pass success [30, 31]. Wave form capnography was not used for 100% of patients, and this is an area that should be improved on, as NAP4 recommendations specify that capnography should be used for all intubated patients [8].

EAM in patients with cardiac arrest might differ from those with return of spontaneous circulation (ROSC) or those with different indications requiring intubation. During cardiac arrest, EAM is performed in a time sensitive manner and as such a checklist may not be used and no induction or paralytic drugs given. We noted that an intubation checklist was used in 48% of medical cardiac arrest and 45% had a paralytic agent. We assume that these patients were intubated post ROSC as we did not collect specific data on pre and post ROSC care for cardiac arrest patients. Anecdotally, some EDs have crash intubation checklists but this data was not included in the present study.

Our study had some limitations. The accuracy of the data was dependent on clinicians entering information simultaneously after intubation. Therefore, CI’s followed up intubating clinicians and reviewed medical records to ensure maximum data capture. Though data was collected by CI’s weekly, we are unable to assure all intubated patients were captured across the 11 EDs participating in NERAA due to local variations and a critical lack of electronic patient records nationally. Reporting bias may have been a potential issue with over reporting of positive findings and under reporting complications. Implementation of the EMARI document for data collection might have led to an improved first pass success rate in our study and improved practices in the participating EDs. The overall sample size of this pilot study was small, hence not adequately powered. Therefore, our analysis and findings should be interpreted with caution. Though our findings may not be generalised to all Irish EDs, it is noteworthy to acknowledge good geographical and ED size representation (Supplement 3).

## Conclusion

This pilot multisite study of EAM in Ireland has shown that most intubations are performed by doctors at registrar level with first pass success and complication rates comparable to international studies. Variation in EAM practice may exist between high and low volume EDs. However, further research is needed to evaluate and establish practice variation between sites in Ireland.

This study was the inaugural ITERN project and showed the impact trainee led research could have on answering important questions related to EAM in Ireland. This study highlights areas for quality improvement in EAM particularly around the use of intubation checklist, video laryngoscopy, bougie and capnography. The importance of clinical registries in airway management are well established and it is anticipated that this study may serve as a platform for development of a national project for ongoing surveillance of EAM in Ireland.

## Supplementary Information

**Additional file 1.**

**Additional file 2.**

**Additional file 3.**

## Data Availability

Data from individual hospitals will be made available to staff in those hospitals. Deidentified data will be made available on request. All requests must be emailed to team@itern.ie and approved by steering committee.

## Abbreviations

BVM: Bag Valve Mask
DAS: Difficult Airway Society
EAM: Emergency airway management
EM: Emergency Medicine
ED: Emergency Department
EMARI: Emergency Medicine Airway Registry Ireland
ICM: Intensive Care Medicine
ICU: Intensive Care Unit
ITERN: Irish Trainee Emergency Research Network
IAEM: Irish Association for Emergency Medicine
ICEMT: Irish College of Emergency Training
LMA: Laryngeal Mask Airway
NEAR: National Emergency Airway Register
NAP4: 4th National Audit Project of the Royal College of Anaesthetists and The Difficult Airway Society
NIV: Non Invasive Ventilation
NCHDs: Non-Consultant Hospital Doctors
NP: Nasal Prongs
PEEP: Positive End Expiratory Pressure
RCEM: Royal College of Emergency Medicine
RSI: Rapid sequence Induction
SHO: Senior House Officer
TERN: Trainee Emergency Research Network UK
