# Supplementary material for: National Emergency Resuscitation Airway Audit (NERAA): a pilot multicentre analysis of emergency intubations in Irish emergency departments
Source: BMC Emerg Med. 2022 May 28;22:91. doi: 10.1186/s12873-022-00644-2 (PMC9148500; doi:10.1186/s12873-022-00644-2)

EMERGENCY MEDICINE AIRWAY REGISTRY - IRELAND

IRISH ASSOCIATION FOR EMERGENCY MEDICINE

HOSPITAL EMARI NUMBER:

PATIENT MRN:

DATE:

TIME:

PATIENT WEIGHT (KG):

WEIGHT ESTIMATED? Y N

INTUBATOR:

SPECIALTY & GRADE:

INDICATION FOR INTUBATION - TICK ALL THAT APPLY

MEDICAL:		TRAUMA:	
Respiratory failure	☐	Traumatic cardiac arrest	☐
Airway obstruction	☐	Shock	☐
Anaphylaxis	☐	Neck / facial trauma	☐
Cardiac failure	☐	Burn / inhalation	☐
Sepsis	☐	Drowning	☐
GI bleed	☐	Chest trauma	☐
Seizure	☐	Penetrating trauma	☐
Altered mental status	☐	Head injury - airway not patent	☐
Overdose / poisoning	☐	Head injury - threatened airway	☐
Cardiac arrest	☐	OTHER:	
Intra-cranial haemorrhage / stroke	☐		

WAS AN AIRWAY ASSESSMENT MADE?	Y N	WAS A DIFFICULT AIRWAY PREDICTED?	Y N
WHAT DIFFICULTIES WERE PREDICTED?	Difficult face-mask ventilation? Y N<br>Other:	Difficult intubation?	Y N

OBSERVATIONS						
	AT TIME OF DECISION TO INTUBATE	GCS	RR	SBP	HR	SaO_2
	FIRST SET AFTER INTUBATION			SBP	HR	SaO_2

PREOX* FINAL DEVICE USED	NRBM ☐	BVM ☐	BVM + PEEP ☐	NIV ☐	LMA ☐	PATIENT POSITION TICK ONE ONLY	Flat ☐	Bed tilted head up ☐
APNOEIC O_2† TICK ALL THAT APPLY	None ☐	NP ☐	BVM ☐	NIV ☐	LMA ☐		Pillow or occipital pad ☐	Ramped or head up ☐

EMERGENCY INTUBATION CHECKLIST COMPLETED	Y N	TIME OF INDUCTION (24HRS)	HH:MM	TIME OF INTUBATION (24HRS)	HH:MM

DRUGS FOR RAPID SEQUENCE INDUCTION

INDUCTION AGENT	DOSE GIVEN	PARALYTIC AGENT	DOSE GIVEN	POST-INTUBATION SEDATION	
Fentanyl	micrograms	Rocuronium	mg	Drug Chosen:	
Propofol	mg	Suxamethonium	mg	Rate:	
Ketamine	mg	Other:			
Thiopentone	mg	NONE ☐			
Etomidate	mg			DELAYED SEQUENCE INDUCTION	
Midazolam	mg			Delayed sequence induction performed?	Y N
Other:				Ketamine (1st dose)	mg
NONE ☐				Ketamine (2nd dose)	mg

*NRBM = Non Re-Breather mask; BVM = Bag Valve Mask; BVM + PEEP = PEEP valve attached to BVM; NIV = Non-Invasive Ventilation; LMA = Laryngeal Mask Airway

†NP = Nasal Prongs; BVM = Active Ventilation using BVM after induction until laryngoscopy; NIV = using NIV after induction until laryngoscopy

ATTEMPT	INTUBATOR	SPECIALITY + GRADE	NUMBER OF PREVIOUS INTUBATIONS (CIRCLE)	LARYNGOSCOPE M=MACINTOSH V=VIDEO O=OTHER	CORMACK & LEHANE (1 / 2 / 3 / 4) DIRECT VISION	CORMACK & LEHANE (1 / 2 / 3 / 4) VIDEO	B=BOUGIE S=STYLET N=NEITHER	EXTERNAL LARYNGEAL MANIPULATION (Y / N)	CRICOID (Y / N)	MANUAL IN-LINE STABILISATION (Y / N)
1			<10, 10-100, >100							
2			<10, 10-100, >100							
3			<10, 10-100, >100							
4			<10, 10-100, >100							
5			<10, 10-100, >100							

INTUBATION MANOEUVRES - TICK ALL THAT APPLY

NONE	☐	Cricoid pressure removed	☐
OPA / NPA inserted post induction	☐	LMA inserted post induction	☐
BVM ventilation after failed attempt	☐	Patient position changed	☐
		Surgical Airway	☐

ETT PLACEMENT CONFIRMATION - TICK ONE ONLY

Waveform capnography	☐	Colour change capnometry	☐	Clinical confirmation alone	☐

INTUBATION COMPLICATIONS - TICK ALL THAT APPLY

NONE	☐	Oesophageal intubation	☐
Equipment failure (state in comments)	☐	Mainstem bronchial intubation	☐
Desaturation - SaO_2 <93%	☐	Vomit - no aspiration	☐
Bradycardia - HR <60bpm	☐	Vomit - with aspiration	☐
Hypotension - requiring IV fluid / pressors	☐	Laryngospasm	☐
Dental trauma due to intubation	☐	Medication error	☐
Airway trauma by intubator	☐	Cardiac arrest	☐
Second dose of paralytic agent	☐	Other (state in comments)	☐

DISPOSITION - TICK ONE ONLY

ICU ☐	Theatre / Cath lab ☐	Transferred to another hospital ☐	Required subsequent intubation in ED ☐	Extubated in ED ☐	Died in ED ☐

COMMENTS

CLASSIFICATION (CIRCLE BOTH)

GRADE I | GRADE II | GRADE III | GRADE IV

MALLAMPATI

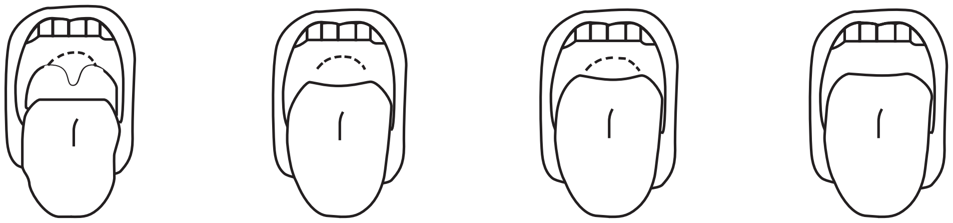

CORMACK & LEHANE

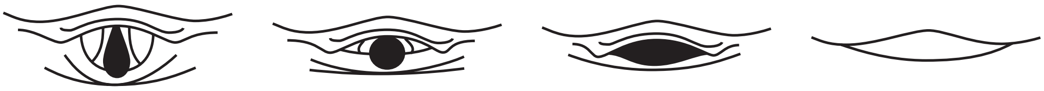

Supplement: Supplementary file 1 — Additional file 1. [file 12873_2022_644_MOESM1_ESM.pdf]
