# Supplementary material for: National Emergency Resuscitation Airway Audit (NERAA): a pilot multicentre analysis of emergency intubations in Irish emergency departments
Source: BMC Emerg Med. 2022 May 28;22:91. doi: 10.1186/s12873-022-00644-2 (PMC9148500; doi:10.1186/s12873-022-00644-2)
Supplement: Supplementary file 2 — Additional file 2. [file 12873_2022_644_MOESM2_ESM.docx]

Supplement 2A Reported EAM per week during study period (March 10 – May 10 2020)

Supplement 2B Emergency Department attendance per month.
