# Supplementary material for: National Emergency Resuscitation Airway Audit (NERAA): a pilot multicentre analysis of emergency intubations in Irish emergency departments
Source: BMC Emerg Med. 2022 May 28;22:91. doi: 10.1186/s12873-022-00644-2 (PMC9148500; doi:10.1186/s12873-022-00644-2)
Supplement: Supplementary file 3 — Additional file 3. [file 12873_2022_644_MOESM3_ESM.pdf]

Supplement 3 NERAA site location and Emergency Department annual attendance

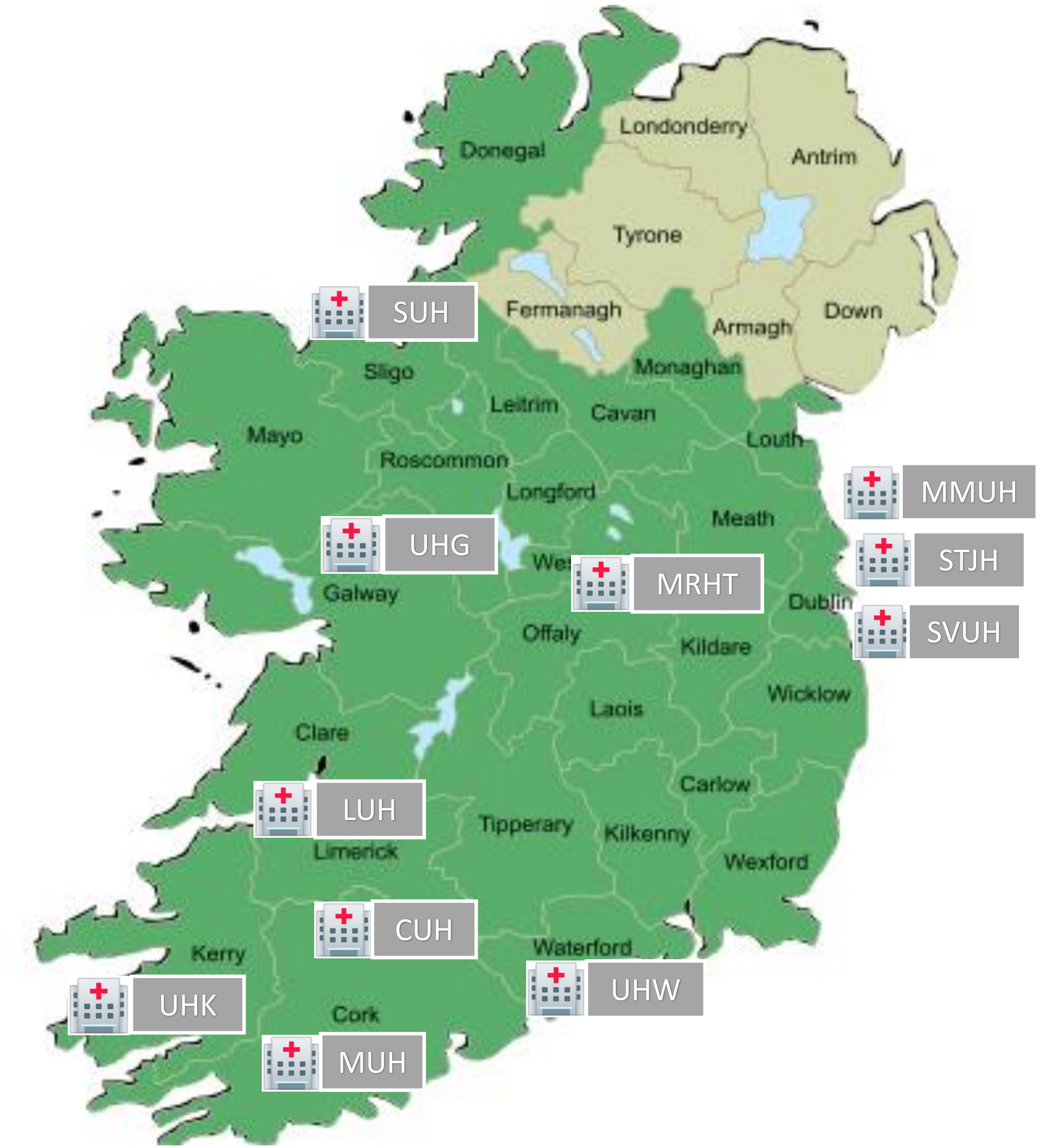

Count	Code	Hospital	Count
1	SUH	Sligo University Hospital	45000
2	MMUH	Mater Misericordiae University Hospital	82000
3	MUH	Mercy University Hospital	25000
4	UHK	University Hospital Kerry	37200
5	UHG	University Hospital Galway	65000
6	SVUH	St Vincent's University Hospital Dublin	60000
7	MRHT	Midland Regional Hospital Tullamore	31000
8	UHW	University Hospital Waterford	50000
9	CUH	Cork University Hospital	70000
10	STJH	St James Hospital Dublin	65000
11	LUH	Limerick University Hospital	71000
